# Biosynthesis of Silver Nanoparticles from Oropharyngeal *Candida glabrata* Isolates and Their Antimicrobial Activity against Clinical Strains of Bacteria and Fungi

**DOI:** 10.3390/nano8080586

**Published:** 2018-08-01

**Authors:** Mohammad Jalal, Mohammad Azam Ansari, Mohammad A. Alzohairy, Syed Ghazanfar Ali, Haris M. Khan, Ahmad Almatroudi, Kashif Raees

**Affiliations:** 1 Department of Microbiology, Jawaharlal Nehru Medical College and Hospital, Aligarh Muslim University, Aligarh 202002, India; syedmicro72@gmail.com (S.G.A.); harismk2003@hotmail.com (H.M.K.); 2 Department of Epidemic Disease Research, Institutes of Research and Medical Consultations (IRMC), Imam Abdulrahman Bin Faisal University, 31441 Dammam, Saudi Arabia; 3 Department of Medical Laboratories, College of applied Medical sciences, Qassim University, Qassim 51431, Saudi Arabia; dr.alzohairy@gmail.com (M.A.A.); aamtrody@qu.edu.sa (A.A.); 4 Department of Applied Chemistry, Aligarh Muslim University, Aligarh 202002, India; raeeskashif@gmail.com

**Keywords:** *Candida glabrata*, extracellular, mycosynthesis, MIC, MBC, MFC, membrane integrity, TEM, FTIR

## Abstract

The objective of the present study was one step extracellular biosynthesis of silver nanoparticles (AgNPs) using supernatant of *Candida glabrata* isolated from oropharyngeal mucosa of human immunodeficiency virus (HIV) patients and evaluation of their antibacterial and antifungal potential against human pathogenic bacteria and fungi. The mycosynthesized AgNPs were characterized by color visualization, ultraviolet-visible (UV) spectroscopy, fourier transform infrared spectroscopy (FTIR), and transmission electron microscopy (TEM). The FTIR spectra revealed the binding and stabilization of nanoparticles with protein. The TEM analysis showed that nanoparticles were well dispersed and predominantly spherical in shape within the size range of 2–15 nm. The antibacterial and antifungal potential of AgNPs were characterized by determining minimum inhibitory concentration (MIC), minimum bactericidal concentration (MBC)/ minimum fungicidal concentration (MFC), and well diffusion methods. The MBC and MFC were found in the range of 62.5–250 μg/mL and 125–500 μg/mL, which revealed that bacterial strains were more susceptible to AgNPs than fungal strains. These differences in bactericidal and fungicidal concentrations of the AgNPs were due to the differences in the cell structure and organization of bacteria and yeast cells. The interaction of AgNPs with *C. albicans* analyzed by TEM showed the penetration of nanoparticles inside the *Candida* cells, which led the formation of “pits” and “pores” that result from the rupturing of the cell wall and membrane. Further, TEM analysis showed that *Candida* cells treated with AgNPs were highly deformed and the cells had shrunken to a greater extent because of their interaction with the fungal cell wall and membrane, which disrupted the structure of the cell membrane and inhibited the normal budding process due to the destruction and loss of membrane integrity and formation of pores that may led to the cell death.

## 1. Introduction

It is worthy to state that the 21st century is the golden era of silver nanotechnology because silver-based materials and silver nanoparticles (AgNPs) are used in textile engineering, waste water treatment and purification, optics, electronics, pharmaceutical industry, as catalysts, optical sensors, and in the medical field as bactericidal, fungicidal, larvicidal, therapeutic, diagnostic, and anticancer agents, in wound dressings, and imaging, etc. [1]. Silver-based materials and AgNPs are also used in the coatings of medical devices, formulation of dental resin composites, coating on water filters, as topical creams to prevent wound infections, as a microbiocidal agent in air sanitizer sprays, respirators, pillows, soaps, laundry detergents, shampoos, socks, wet wipes, washing machines, wall paints, toothpastes, etc. [1,2,3].

Due to the broad application of AgNPs in various fields, as mentioned above, the demand for large-scale, eco-friendly, non-hazardous, faster, and biocompatible production of AgNPs with specific size, shape, and properties have also been increased. AgNPs can be prepared either by physical, chemical, or biological approaches. The synthesis of AgNPs by physical and chemical methods usually requires expensive equipment, high pressure and temperature, stabilizers, and toxic reducing reagents.

The green routes of synthesis of AgNPs by exploring plant parts and microorganisms, such as bacteria, fungi, algae, etc., has advantages over physical and chemical methods as green approaches are environmentally-friendly, cost-effective, fast, pollution-free, and most importantly, provides non-toxic and biocompatible natural reducing and stabilizing agents. Biosynthesis of AgNPs using various species of filamentous fungi, such as species of *Aspergillus*, *Penicillium*, *Fusarium*, *Trichoderma*, *Verticillium*, *Rhizopus*, *Colletotrichum*, and *Neurospora*, have been well studied and documented [4,5]. Nevertheless, reports on biosynthesis of AgNPs using single-celled yeasts remains limited and a few yeast species, such as yeast strain MKY3 [6], *Saccharomyces boulardii* [7], *S. cerevisiae* [8], *Candida albicans* [9,10], *Candida utilis* [11], and *Candida lusitaniae* [5] have been reported.

Yeast has been chosen for the extracellular biosynthesis of AgNPs because of their better tolerance and metal bioaccumulation property, large scale production, economic viability, convenient downstream processing, and most importantly, fungi produces huge amounts of proteins and enzymes that acts as reducing and stabilizing agents [3,12,13,14].

The aims of the present work was to achieve: (i) extracellular mycosynthesis of AgNPs using the supernatant of *Candida glabrata* for the first time as reducing and stabilizing agents; (ii) the characterization of mycosynthesized AgNPs using a ultraviolet- visible (UV-Vis) spectrophotometer, fourier transform infrared (FTIR), and transmission electron microscopy (TEM); (iii) the assessment of antibacterial and anticandidal activity of mycosynthesized AgNPs against *Escherichia coli*, *Salmonella typhimurium*, *Klebsiella pneomonaie*, *Shigella flexneri*, *Pseudomonas aeruginosa*, *Staphylococcus aureus*, *C. albicans*, *C. dubliniensis*, *C. parapsilosis*, *C. tropicalis*, *C. krusei*, and *C. glabrata* using agar well diffusion and standard microdilution methods by determining minimum inhibitory concentration (MIC), minimum bactericidal concentration (MBC), and minimum fungicidal concentration (MFC); and (iv) the investigation of a mode of action of AgNPs against *C. albicans* using transmission lectron microscopy (TEM).

## 2. Materials and Method

### 2.1. Preparation of Candida Glabrata Supernatant for the Synthesis of Silver Nanoparticles

*Candida glabrata* were isolated from the oropharyngeal mucosa of patients having oral candidiasis by using a sterile cotton swab. The swabs were then dipped into 5 mL of Sabouraud dextrose broth (SDB) (HiMedia, Mumbai, India) and then 0.1 mL of culture was spread homogenously onto the HiCHROM differential agar (HiMedia, Mumbai India) plates at 37 °C for 48 h. *Candida* spp. were identified by morphology on corn meal agar (CMA), sugar assimilation (SAT), HiCrome agar, and a germ tube test [15]. *C. glabrata* isolates developed characteristic cream to white colonies, whereas *C. albicans* isolates formed light-green colonies. Further, trehalase and maltase tests were performed to confirm the *C. glabrata* isolates [16]. One to two pure colonies of fresh *C. glabrata* cultures were again suspended into 500 mL SDB and incubated for 48–72 h at 28 °C. After incubation, the culture was centrifuged at 12,000 rpm for 10 min and the supernatant was collected and stored at 4 °C for the synthesis of AgNPs. All media was purchased from HiMedia, Mumbai, India.

### 2.2. Extracellular Mycosynthesis, Sepration and Purification of AgNPs

For the biosynthesis of AgNPs, 20 mL of collected supernatant was added in 1 mM of silver nitrate and then the solution was kept at room temperature overnight. The reduction of silver nitrate into AgNPs showed a change in color of silver nitrate from colorless to brown, which is an indication of the formation of AgNPs. Further, the synthesized AgNPs were separated and isolated by ultracentrifugation as previously described with slight modification [17,18]. To remove excess, unreduced silver ions and other possible impurities from the supernatant, the reaction mixture containing NPs were centrifuged at 14,000 rpm for 20 min with Milli-Q water. The process of centrifugation was repeated at least four times to ensure a better separation of NPs [17]. The particles separated were then resuspended again in ethanol and then centrifugation was further repeated three times [19]. A dark sediment was formed at the bottom of the centrifugal tube. After that, a dried powder of the AgNPs was obtained by freeze-drying [18], and their characterization was performed.

### 2.3. Characterization of Silver Nanoparticles

**Figure A1:**
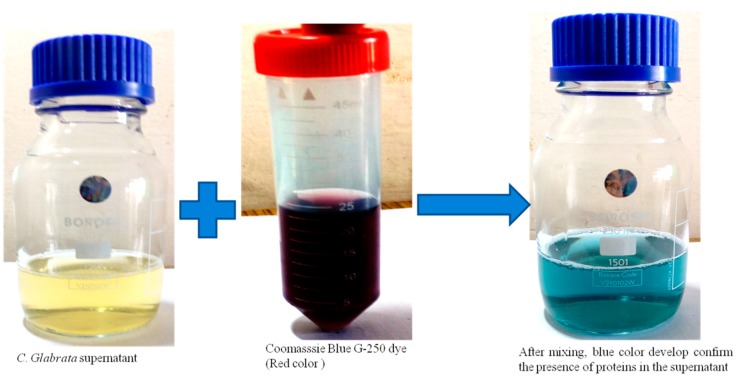
A simple protein-dye binding assay to assess the presence of protein in the supernatant.

The biosynthesized AgNPs were preliminarily characterized by visual observation of the color change of the reaction mixture and then by measuring the absorbance of the colloidal suspension before and after the isolation NPs from the supernatant using UV-Vis spectrophotometer (Perkin-Elmer Lambda 25, Shelton, CT, USA). The preparation of samples for absorbance was accomplished as described in our previous work [20]. Briefly, AgNPs were subjected to mild sonication for 20 min, after which the UV-Vis spectra of AgNPs was monitored in the range of 270–700 nm. Distilled water was used to adjust the baseline [20]. Further, to confirm the presence of protein in the supernatant and their possible role in synthesis and reduction of AgNO_3_ was carried out by a simple protein-dye binding assay, i.e., Bradford assay (Figure A1). The functional group present in the supernatant that was responsible for the synthesis of AgNPs was analyzed by FTIR (SHIMADZU-8400 spectrometer, Tokyo, Japan) in the range of 400 to 4000/cm. Further, the surface morphology, shape, and size of synthesized AgNPs were characterized by transmission electron microscopy (Joel 2100, Tokyo, Japan).

### 2.4. Tested Microorganisms

The clinical isolates of fungal and bacterial species, i.e., *C. albicans*, *C. tropicalis*, *C. parapsilosis*, *C. dubliniensis*, *C. krusei*, *C. glabrata*, *S. aureus*, *E. coli*, *P. aeruginosa*, *K. pneomonaie*, *S. typhimurium*, and *S. flexneri* isolated from the oropharyngeal mucosa of the patients used in this study were obtained from the Department of Microbiology, Jawaharlal Nehru Medical College and Hospital, Aligarh Muslim University, India.

### 2.5. Evaluation of Antimicrobial Activity of Mycosynthesized AgNPs Using a Diffusion Method

The antimicrobial activity of as-synthesized AgNPs against various bacterial isolates, e.g., *S. aureus*, *E. coli*, *S. typhimurium*, *S. flexneri*, *K. pneomonaie*, *P. aeruginosa*, and *Candida* spp., e.g., *C. albicans*, *C. dubliniensis*, *C. parapsilosis*, *C. tropicalis*, *C. krusei*, and *C. glabrata* were examined using a well diffusion method as previously reported [17]. Briefly, a 6 mm diameter well was made on Nutrient and Sabouraud dextrose agar plates previously inoculated with 100 μL of 1 × 10^6^ bacterial and fungal suspension, and then varying concentrations of AgNPs were aseptically filled in the well. The plates were subsequently incubated at 37 °C (Bacteria) and 28 °C (*Candida* spp.) for 24 h, and the antimicrobial activity was analyzed by measuring the diameter of the inhibition zone (mm) around the well.

### 2.6. Assessment of Antimicrobial Activity of AgNPs by Determining MIC and MBC/MFC

Minimal inhibitory concentration (MIC): The MIC values of mycosynthesized AgNPs against bacterial and *Candida* isolates were assessed using the method described by Ansari et al. [21].

Minimal bactericidal and fungicidal concentration (MBC/MFC): Further, MBC and MFC values of AgNPs against all tested bacteria and *Candida* spp. was examined using a method described in References [17,21].

### 2.7. Ultrastructural Morphological Changes Caused by AgNPs in C. albicans: TEM Analysis

The morphological and ultrastructure alteration caused by AgNPs in *C. albicans* cells were examined by using transmission electron microscope. The sample preparation and analysis procedures were similar to those described in our previous research work [17].

## 3. Results and Discussion 

### 3.1. Characterization of Biosynthesized AgNPs

**Figure 1:**
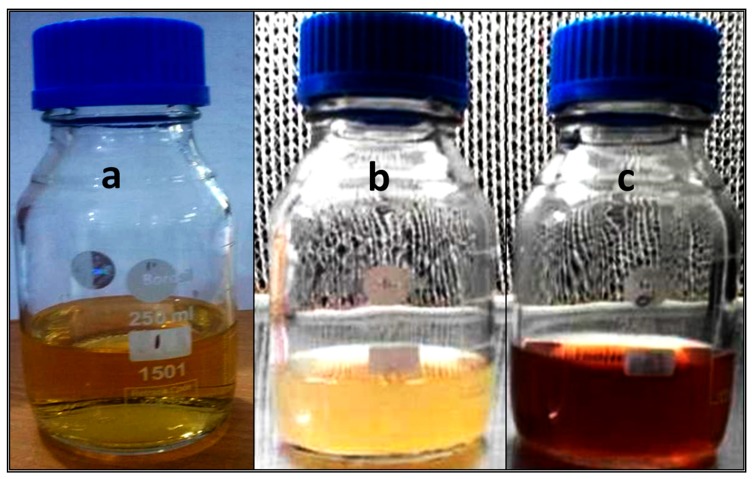
Biosynthesis of AgNPs using an extracellular filtrate of *C. glabrata* after 12 h at room temperature in dark conditions. (**a**) Sterile Sabouraud dextrose broth with AgNO_3_; (**b**) Cell filtrate without AgNO_3_; (**c**) Cell filtrate with AgNO_3._

**Figure 2:**
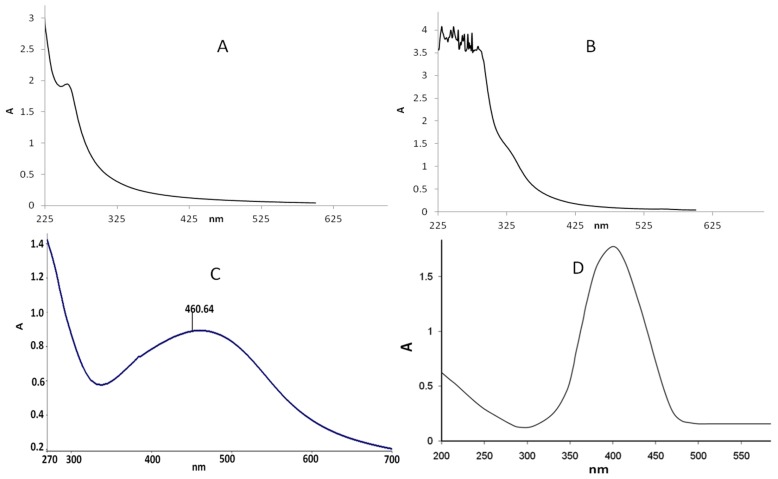
(**A**) UV-visible spectra of 1 mM AgNO_3_; (**B**) *C. glabrata* supernatant before the addition of AgNO_3_; (**C**) after addition AgNO_3_ (biosynthesized AgNPs); and (**D**) commercially available spectra of AgNPs of known size, i.e., 5–16 nm [20].

In the present study, *C. glabrata* supernatant was used for the synthesis of AgNPs. After the addition of *C. glabrata* supernatant with 1 mM aqueous solution of silver nitrate, a brownish color was observed, which indicated the extracellular biosynthesis of AgNPs (Figure 1). Previously, it has been reported that the reduction of Ag^+^ into AgNPs can be seen very clearly when the color of the solution changes from colorless to brown due to the surface plasmon resonance [6]. Figure 2 shows the UV-Vis spectroscopy of the as-prepared AgNPs in the range of 270–700 nm and a single strong peak was observed at 460.64 nm, which is the characteristic of AgNPs. Bhat et al. [10] reported that the absorption line of biosynthesized AgNPs was 430 nm when the silver nitrate solution was challenged with *C. albicans*.

**Figure 3:**
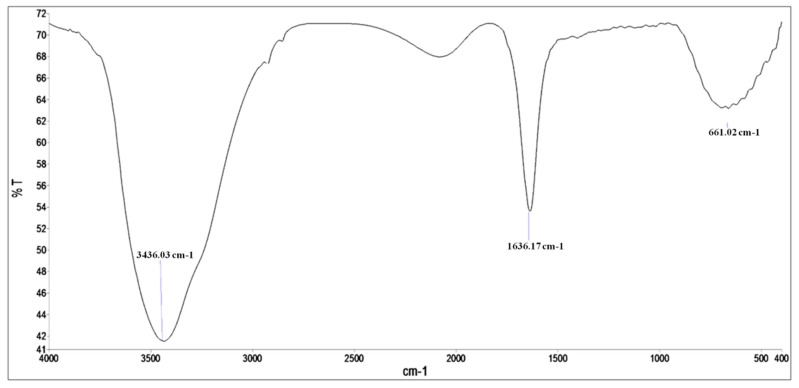
FTIR spectra of AgNPs synthesized by the *C. glabrata* supernatant.

FTIR analysis was performed to identify the potential biomolecules and functional groups responsible for the reduction of silver ions to Ag^0^ and stabilization of AgNPs [22]. Figure 3 shows a strong absorption line at 3436.03 cm^−1^, which corresponds to the –OH groups that could arise from carbohydrates present in the supernatant, and the peak at 1636.17 cm^−1^ is due to amides (C–O stretch), i.e., characteristic of the presence of protein and enzymes in the supernatant that confirm the extracellular formation of AgNPs [23]. The peak at 661 cm^−1^ corresponds to C–H (alkane) and C=H bonding (alkene). In a previous study, Bhat et al. [10] found that the protein biomolecules present in the extract of *C. albicans* bind to the synthesized nanoparticles either by free amino or carboxyl groups, and the released extracellular proteins could possibility stabilize the biosynthesized AgNPs.

The mechanism of biosynthesis of AgNPs using microorganisms are well understood. It was reported that the biomass or supernatant of microorganisms possibly act as bioreductant and capping agents due to the presence of biomolecules such as proteins, amino acids, enzymes, vitamins, and polysaccharides [10,23]. However, the most widely accepted mechanism for the biosynthesis of AgNPs and other nanoparticles using microbes is mainly due to the presence of enzyme Nicotinamide adenine dinucleotide (NADH) and NADH-dependent nitrate reductase [24,25,26]. The role of nitrate reductase in the synthesis of AgNPs has been demonstrated by exploring the purified nitrate reductase in vitro for the synthesis of AgNPs and it was found that the reduction of silver ions happens by means of the transfer of electrons from NADH where enzyme NADH-dependent reductase acts as a carrier [24,25,26].

**Figure 4:**
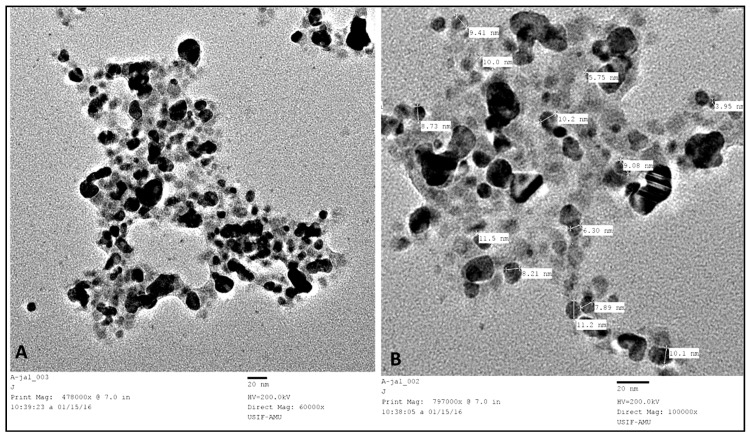
TEM images of AgNPs synthesized by *C. glabrata* supernatant. (**A**): At lower magnification (60,000×); (**B**): at higher magnification (100,000×).

The TEM micrograph of as-prepared AgNPs exhibited that the synthesized AgNPs were predominantly spherical and oval, well-dispersed, and uniform with a size range of 2–15 nm (Figure 4). Niknejad et al. [8] reported biosynthesis of AgNPs using the yeast *S. cerevisiae* and they found that AgNPs were mainly spherical and polydispersed within the size of 5–20 nm. The size of AgNPs obtained in our study was much smaller than AgNPs synthesized from *Candida utilis*, where the size was 20–80 nm [11]. The image shows both individual and aggregated AgNPs (Figure 4). The particles were well-dispersed and not in direct contact due to capping and stabilization of AgNPs by the proteins around the periphery of the nanoparticles (Figure 4), which further confer the involvement of extracellular proteins and enzymes secreted by *C. glabrata* in the supernatant that acted as reducing and capping agents [22].

### 3.2. Antimicrobial Activity of Biosynthesized AgNPs

**Figure 5:**
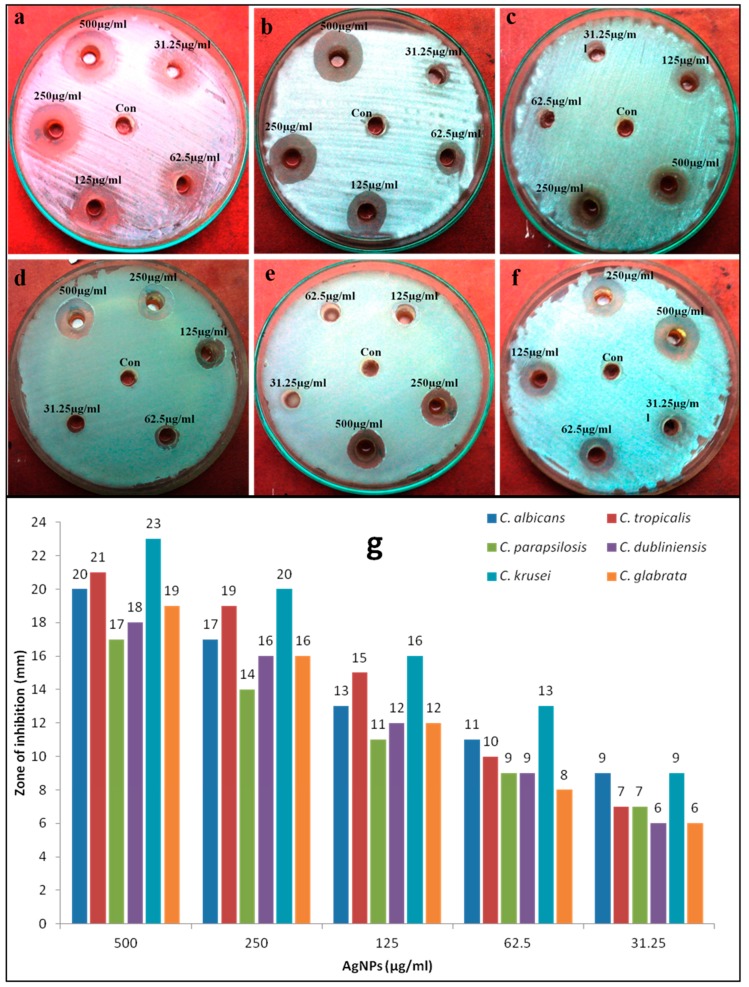
Anticandidal activity of mycosynthesized AgNPs at different concentrations evaluated by agar well diffusion method against different Candida spp.: (**a**) *C. albicans*, (**b**) *C. tropicalis*, (**c**) *C. dubliniensis*, (**d**) *C. glabrata*, (**e**) *C. parapsilosis*, and (**f**) *C. krusei*. Panel (**g**) represents the zone of inhibition (in mm) at different concentrations of AgNPs.

**Figure 6:**
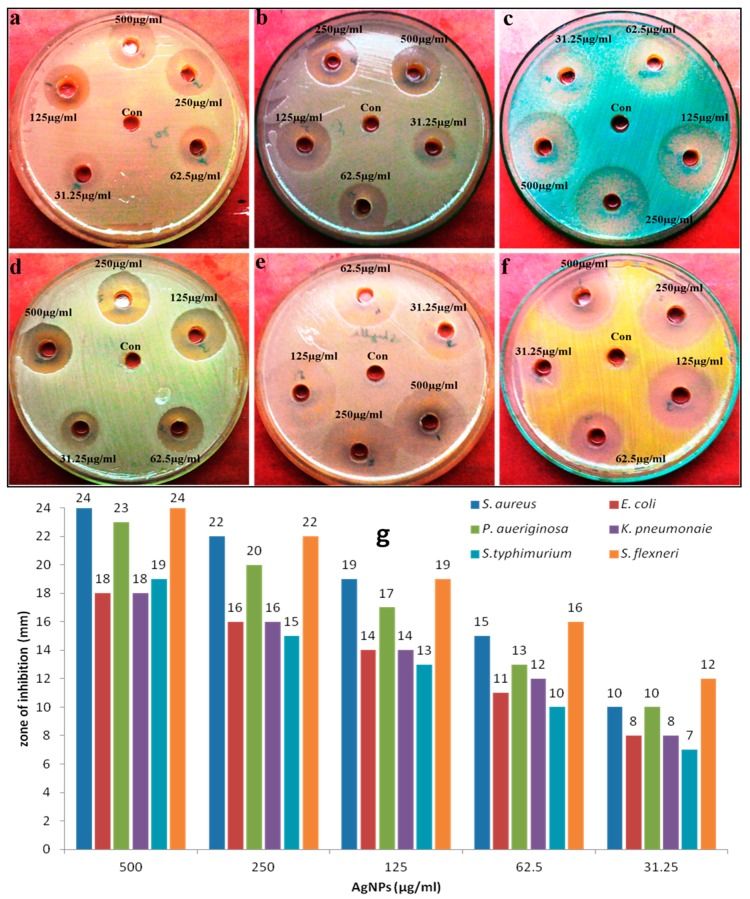
Antibacterial activity of mycosynthesized AgNPs at different concentrations evaluated by agar well diffusion method against different pathogenic bacteria: (**a**) *E. coli*, (**b**) *K. pneomonaie*, (**c**) *P. aeruginosa*, (**d**) *S. typhimurium*, (**e**) *S. flexneri*, and (**f**) *S. aureus*. Panel (**g**) represents the zone of inhibition (in mm) at different concentrations of AgNPs.

Antimicrobial activity of biosynthesized AgNPs against human pathogenic microorganisms, i.e., *Candidal* spp. and bacterial spp. was evaluated by agar well diffusion and two-fold microdilution methods. A zone of inhibition test of mycosynthesized AgNPs against tested bacteria and *Candida* spp. at different concentrations are shown in Figure 5 and Figure 6, and it was very clear that AgNPs had an excellent inhibition zone against all strains. The inhibition zone diameter showed that AgNPs were more effective against bacterial strains in comparison to fungi (Figure 5 and Figure 6). No antimicrobial activity has been observed by the *C. glabrata* supernatant. Further, the antimicrobial properties of AgNPs against various bacterial and fungal strains were examined by determining the MIC, MBC, and MFC. The MIC and MBC values for all tested bacterial strains were found in the range of 31.25–125 μg/mL and 62.5–250 μg/mL, respectively (Table 1), whereas the MIC and MFC values for fungal strain was in the range of 62.5–250 μg/mL and 125–500 μg/mL, respectively (Table 2). It was found that AgNPs were bactericidal at low concentration (62.5–250 μg/mL) and fungicidal at high concentration (125–500 μg/mL), which revealed that bacterial strains were more susceptible to AgNPs than fungal strains. In general, it was found that gram-negative and gram-positive bacterial strains showed better antimicrobial activity when compared to fungi *Candida* spp. (Table 1 and Table 2). These differences in bactericidal and fungicidal concentrations of the AgNPs were due to the differences in the cell structure and organization of the bacteria and yeast cells. The bacterial cell structure is less complex and they are evolutionarily prokaryotic types, and were therefore unable to fight the toxic effects of AgNPs as effectively as the eukaryotic yeast cells that can resist higher concentrations of AgNPs because of their better cell organization and much more complex structure, and superior detoxification system [27].

### 3.3. Ultrastructural Changes in C. albicans after Exposure to AgNPs: TEM Analysis

**Figure 7:**
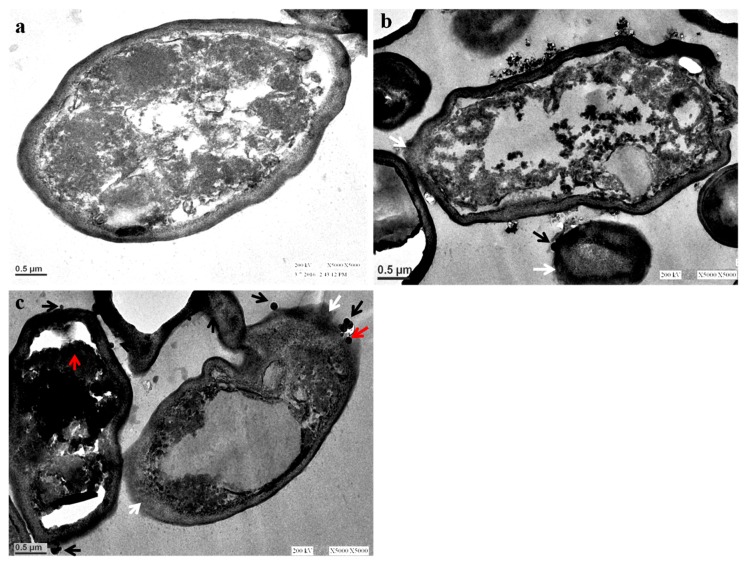
TEM images of *C. albicans*. (**a**) Untreated control cells; (**b**,**c**) Cells treated with 250 µg/mL and 500 µg/mL of AgNPs showing the attachment (black arrows) and penetration of AgNPs inside the cells (red arrows), degradation, destruction, and separation of the outer-most layers of the cell wall and cytoplasmic membrane (white arrows).

The morphological alteration after exposure to biosynthesized AgNPs in *C. albicans* was investigated using a transmission electron microscope. TEM analysis clearly showed that the untreated *C. albicans* exhibited a normal and well-conserved cell wall that was mainly composed of an outer layer, an intermediate space, and a thin innermost layer of the cell membrane (Figure 7a). However, the *Candida* cells after the AgNP treatment showed an aberrant morphological structure and severe damage that was characterized by the formation of “pits” and “pores” that result in the rupturing of the cell wall and membrane (Figure 7b,c). *Candida* cells treated with AgNPs were highly deformed and the cells had shrunken to a great extent (Figure 7b,c). Further, it was observed that the damaged cells exhibited either complete or localized separation of the membrane from the cell wall (Figure 7b,c). Nasrollahi et al. [28] reported that the antifungal activity of AgNPs was due to the formation of “pits” and “pores” on the surfaces of *C. albicans* and *S. cerevisiae* cells that led to the cell death. It has been observed that AgNPs not only anchor to cells at several sites (Figure 7b,c; black arrows), but they penetrate inside the cells (Figure 7c; red arrows), which could result in cell lysis. In their study, Vazquez-Muñoz et al. found that AgNPs were non-specifically distributed in different regions of the cytoplasm and cell wall, and the mode of action of AgNPs was due to the releasing of silver ions that induced cell death [29]. In the present study, it was observed that the rupturing, disintegration, and detachment of the cell wall and membrane from the cells led to the death of *C. albicans* (Figure 7b,c). Kim et al. found that AgNPs exert an antifungal activity because of their interaction with the fungal cell wall and membrane, which disrupts the structure of the cell membrane and inhibits the normal budding process due to the destruction and loss of membrane integrity, and due to the formation of pores that may led to the cell death [30]. Ishida et al. [31] also reported a similar mode of action of AgNPs against yeast *Cryptococcus neoformans* where they found that the antifungal activity of AgNPs was due to the disruption of the cell wall and cytoplasmic membrane [31]. Hwang et al. [32] found that AgNPs exert an antifungal activity against *C. albicans* due to the production and accumulation of reactive oxygen species (ROS) and free hydroxyl radicals (^•^OH) inside the cells, which regulates and induces the cell death through mitochondrial dysfunctional apoptosis, release of cytochrome *c*, nuclear fragmentation, DNA damage, and the activation of metacaspases [32].

## 4. Conclusions 

In this present study, a simple, one-step, safe, rapid, pollutant free, cost-effective, and ecofriendly extracellular silver nanoparticles were prepared using the supernatant of yeast *C. glabrata* as reducing and stabilizing agents for the first time. The synthesized nanoparticles were characterized by UV-visible, TEM, and FTIR spectra. Extracellular synthesis of AgNPs by yeast, i.e., *C. glabrate*, has advantages over mycelium fungi because separation of the particles is much easier and simpler, takes less time without much sophistication, and therefore, extracellular synthesis of AgNPs has importance from the point of view of large-scale production in an environmentally-friendly approach. The extracellular mycosynthesized AgNPs showed excellent antibacterial and antifungal activity against gram-negative and gram-positive pathogenic bacteria and *Candida* spp., respectively. Further, the interaction of AgNPs with *C. albicans* studied by TEM showed the primary attachment and penetration of nanoparticles inside the *Candida* cell that caused the rupturing, disintegration, and detachment of the cell wall and membrane from the cells that led to the death of *C. albicans*. Thus, these mycosynthesized AgNPs may lead to the development of appropriate pharmaceuticals and represent an alternative remedy for the treatment of bacterial and fungal infections, but this needs further in vivo cytotoxic studies before being brought into the market.

## Figures and Tables

**Table 1:** MIC and MBC values of biosynthesized AgNPs against bacterial strains.

Bacterial Isolates	MIC (µg/mL)	MBC (µg/mL)
*Staphylococcus aureus*	31	62
*Escherichia coli*	31	62
*Pseudomonas aeruginosa*	62	125
*Klebsiella pneomonaie*	62	125
*Salmonella typhimurium*	125	250
*Shigella flexneri*	62	125

**Table 2:** MIC and MFC values of biosynthesized AgNPs against *Candida strains*.

Fungal Isolates	MIC (µg/mL)	MFC (µg/mL)
*Candia albicans*	62	125
*Candida tropicalis*	250	500
*Candida parapsilosis*	250	500
*Candida dubliniensis*	125	250
*Candida krusei*	125	250
*Candida glabrata*	250	500

## References

[1] Prabhu S., Poulose E.K. (2012). Silver nanoparticles: mechanism of antimicrobial action, synthesis, medical applications, and toxicity effects. Int. Nano Lett..

[2] Firdhouse M.J., Lalitha P. (2015). Biosynthesis of silver nanoparticles and its applications. J. Nanotechnol..

[3] Velhal S.G., Kulkarni S.D., Latpate R.V. (2016). Fungal mediated silver nanoparticle synthesis using robust experimental design and its application in cotton fabric. Int. Nano Lett..

[4] Moghaddam A.B., Namvar F., Moniri M., Tahir M.P., Azizi S., Mohamad R. (2015). Nanoparticles biosynthesized by fungi and yeast: a review of their preparation, properties, and medical applications. Molecules.

[5] Eugenio M., Müller N., Frasés S., Almeida-Paes R., Lima L.M., Lemgruber L., Farina M., de Souza W., Sant’Anna C. (2016). Yeast-derived biosynthesis of silver/silver chloride nanoparticles and their antiproliferative activity against bacteria. RSC Adv..

[6] Kowshik M., Ashtaputre S., Kharrazi S., Vogel W., Urban J., Kulkarni S.K., Paknikar K.M. (2002). Extracellular synthesis of silver nanoparticles by a silver-tolerant yeast strain MKY3. Nanotechnology.

[7] Kaler A., Jain S., Banerjee U.C. (2013). Green and rapid synthesis of anticancerous silver nanoparticles by *Saccharomyces boulardii* and insight into mechanism of nanoparticle synthesis. Biomed Res. Int..

[8] Niknejad F., Nabili M., Ghazvini R.D., Moazeni M. (2015). Green synthesis of silver nanoparticles: Advantages of the yeast *Saccharomyces* cerevisiae model. Curr. Med. Mycol..

[9] Atef A.H., Mogda K.M., Mahmoudc H.H. (2013). Biosynthesis of silver nanoparticles (AgNps) (a model of metals) by *Candida albicans* and its antifungal activity on Some fungal pathogens (*Trichophyton mentagrophytes* and *Candida albicans*). N. Y. Sci. J..

[10] Bhat M.A., Nayak B.K., Nanda A. (2015). Evaluation of bactericidal activity of biologically synthesised silver nanoparticles from *Candida albicans* in combination with ciprofloxacin. Mater. Today Proc..

[11] Waghmare S.R., Mulla M.N., Marathe S.R., Sonawane K.D. (2015). Ecofriendly production of silver nanoparticles using *Candida utilis* and its mechanistic action against pathogenic microorganisms. 3 Biotech.

[12] Azizi S., Ahmad M.B., Namvar F., Mohamad R. (2014). Green biosynthesis and characterization of zinc oxide nanoparticles using brown marine macroalga Sargassum muticum aqueous extract. Mater. Lett..

[13] Pati R., Mehta R.K., Mohanty S., Padhi A., Sengupta M., Vaseeharan B., Goswami C., Sonawane A. (2014). Topical application of zinc oxide nanoparticles reduces bacterial skin infection in mice and exhibits antibacterial activity by inducing oxidative stress response and cell membrane disintegration in macrophages. Nanomedicine.

[14] Agarwal H., Kumar S.V., Rajeshkumar S. (2017). A review on green synthesis of zinc oxide nanoparticles-an eco-friendly approach. Resour.-Effic. Technol..

[15] Shettar S.K., Patil A.B., Nadagir S.D., Shepur T.A., Mythri B.A., Gadadavar S. (2012). Evaluation of HiCrome differential agar for speciation of candida. J. Acad. Med. Sci..

[16] Freydiere A.M., Parant F., Noel-Baron F., Crepy M., Treny A., Raberin H., Davidson A., Odds F.C. (2002). Identification of *Candida glabrata* by a 30-second trehalase test. J. Clin. Microbiol..

[17] Jalal M., Ansari M.A., Shukla A.K., Ali S.G., Khan H.M., Pal R., Alam J., Cameotra S.S. (2016). Anticandidal green synthesis and antifungal activity of Al2O3 NPs against fluconazole-resistant *Candida* spp isolated from a tertiary care hospital. RSC Adv..

[18] Liu X., Kang J., Liu B., Yang J. (2018). Separation of gold nanowires and nanoparticles through a facile process of centrifugation. Sep. Purif. Technol..

[19] Yu W., Xie H., Chen L., Li Y., Zhang C. (2009). Synthesis and characterization of monodispersed copper colloids in polar solvents. Nanoscale Res. Lett..

[20] Ashraf J.M., Ansari M.A., Choi I., Khan H.M., Alzohairy M.A. (2014). Antiglycating potential of gum arabic capped-silver nanoparticles. Appl. Biochem. Biotechnol..

[21] Ansari M.A., Khan H.M., Khan A.A., Sultan A., Azam A., Shahid M., Shujatullah F. (2011). Antibacterial activity of silver nanoparticles dispersion against MSSA and MRSA isolated from wounds in a tertiary care hospital of North India. Int. J. Appl. Biol. Pharm. Technol..

[22] Sanghi R., Verma P. (2009). Biomimetic synthesis and characterisation of protein capped silver nanoparticles. Bioresour. Technol..

[23] Gudikandula K., Vadapally P., Charya M.S. (2017). Biogenic synthesis of silver nanoparticles from white rot fungi: Their characterization and antibacterial studies. OpenNano.

[24] Ahmad A., Mukherjee P., Senapati S., Mandal D., Khan M.I., Kumar R., Sastry M. (2003). Extracellular biosynthesis of silver nanoparticles using the fungus *Fusarium oxysporum*. Colloids Surf. B.

[25] Kumar S.A., Abyaneh M.K., Gosavi S.W., Kulkarni S.K., Pasricha R., Ahmad A., Khan M.I. (2007). Nitrate reductase-mediated synthesis of silver nanoparticles from AgNO3. Biotechnol. Lett..

[26] Kalimuthu K., Babu R.S., Venkataraman D., Bilal M., Gurunathan S. (2008). Biosynthesis of silver nanocrystals by *Bacillus licheniformis*. Colloids Surf. B.

[27] Panáček A., Kolář M., Večeřová R., Prucek R., Soukupová J., Kryštof V., Hamal P., Zbořil R., Kvítek L. (2009). Antifungal activity of silver nanoparticles against *Candida* spp.. Biomaterials.

[28] Nasrollahi A., Pourshamsian K.H., Mansourkiaee P. (2011). Antifungal activity of silver nanoparticles on some of fungi. Int. J. Nano Dimens..

[29] Vazquez-Muñoz R., Avalos-Borja M., Castro-Longoria E. (2014). Ultrastructural analysis of *Candida albicans* when exposed to silver nanoparticles. PLoS ONE.

[30] Kim K.J., Sung W.S., Suh B.K., Moon S.K., Choi J.S., Kim J.G., Lee D.G. (2009). Antifungal activity and mode of action of silver nano-particles on *Candida albicans*. BioMetals.

[31] Ishida K., Cipriano T.F., Rocha G.M., Weissmüller G., Gomes F., Miranda K., Rozental S. (2014). Silver nanoparticle production by the fungus *Fusarium oxysporum*: Nanoparticle characterisation and analysis of antifungal activity against pathogenic yeasts. Mem. Inst. Oswaldo Cruz.

[32] Hwang I.S., Lee J., Hwang J.H., Kim K.J., Lee D.G. (2012). Silver nanoparticles induce apoptotic cell death in *Candida albicans* through the increase of hydroxyl radicals. FEBS J..

